# Metabolic responses to cold and warm extremes in the ocean

**DOI:** 10.1371/journal.pbio.3002479

**Published:** 2024-01-17

**Authors:** Juan G. Rubalcaba

**Affiliations:** Department of Biodiversity, Ecology and Evolution, Faculty of Biological sciences, The Complutense University of Madrid, Madrid, Spain

## Abstract

Temperature defines the geographical distribution of species, but its mechanisms are much debated. This Primer explores a new PLOS Biology study which suggests that metabolic constrains can arise in both warm and cold waters, defining geographical range limits of marine species.

## Teaser

Temperature influences the geographical distribution of species, but its mechanisms are much debated. A new study in *PLOS Biology* suggests that metabolic constrains can arise in both warm and cold waters at the geographical range limits of marine species.

Anthropogenic climate warming is causing global changes in the abundance and geographical distribution of marine species worldwide [1]. These trends are primarily driven by the increase in water temperature, which affects physiological performance and fitness and ultimately influences where species can live. Yet, predicting the complex responses of organisms to changes in water temperature remains a major challenge for ecological research. To accurately predict these responses, biologists try to understand the mechanisms linking physiology and environmental conditions. An emerging body of research aims to reproduce these mechanisms using models that describe the physiological responses to temperature fluctuations across space and time—an approach that has the potential to revolutionize the way we predict the global redistribution of species in a warming world [2,3].

Water temperature fundamentally affects aerobic metabolism responsible for producing energy fuelling physiological processes, activity, and reproduction. As water temperature increases, the metabolic rate of many aquatic animals rises exponentially, requiring a concomitant increase in oxygen supply. However, the availability of oxygen in water is limited and diffusion rates of oxygen molecules from the environment to the body are slower in water than in air [4]. Therefore, some organisms may not be able to obtain oxygen at the rate required to meet demand in warming waters. This imbalance between oxygen supply and demand may be especially important for large, active species, and it is predicted to impair their physiological performance in the future [5].

While most research has focused on the metabolic responses to higher temperatures—relevant to anthropogenic climate change—the effect of cold on metabolism has received much less attention. At low temperatures, muscular function is constrained limiting the ability of individuals to forage, hunt, or escape from predators. In addition, the circulatory and ventilatory systems become less effective in the cold, which may reduce the organism’s capacity to supply oxygen to metabolizing tissues [6] Therefore, as in the hot extreme, low temperatures could alter the delicate balance between oxygen supply and demand, limiting metabolic function. This idea suggests that oxygen limitation can explain the temperature dependence of metabolism at both hot and cold extremes. Now, writing in *PLOS Biology*, Endress and colleagues [7] find support for this idea and provide a new mechanistic framework to model aerobic constraints at both hot and cold temperatures.

**Fig 1:**
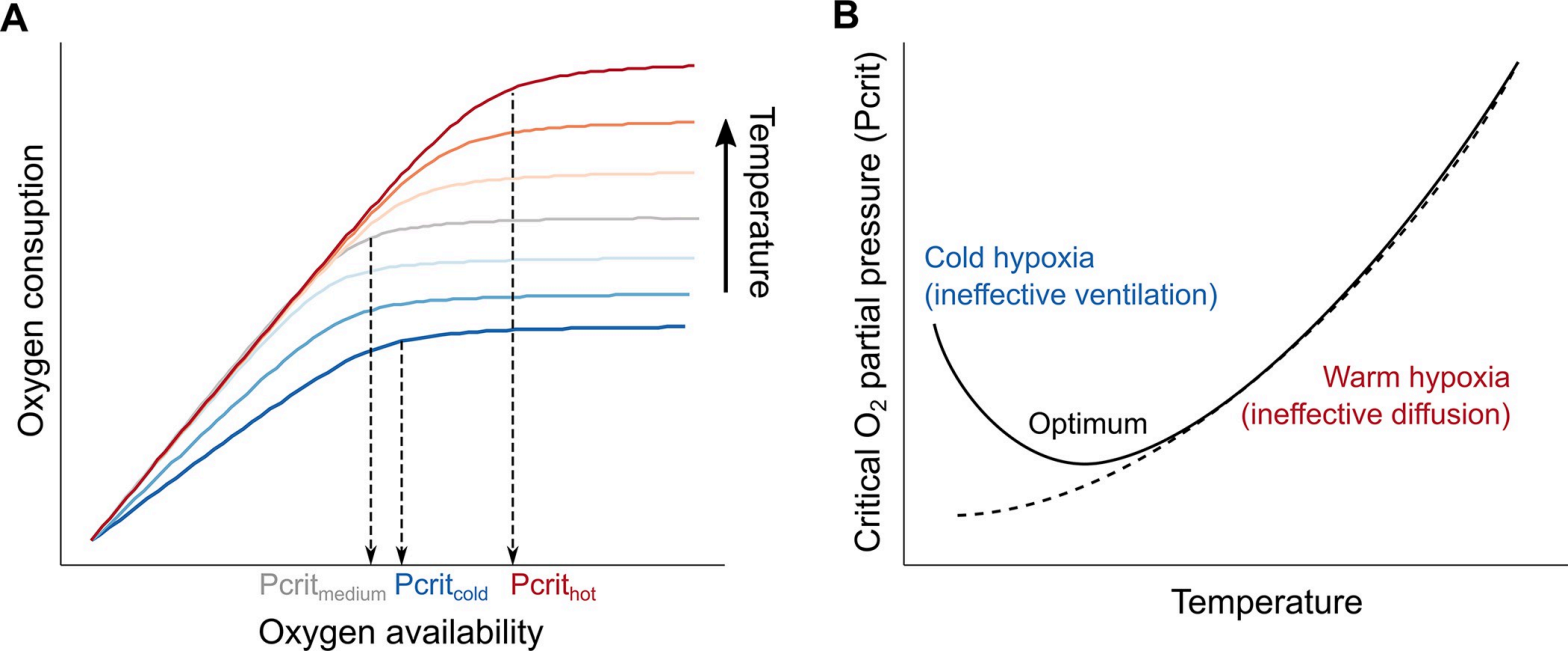
Optimal physiological temperature resulting from the metabolism–temperature–oxygen triad. Oxygen consumption (metabolic rate) draws complex patterns in relation to temperature and environmental oxygen availability: Metabolic rate increases with temperature and the availability of oxygen limits this increase. The minimum oxygen level required to sustain metabolism is called Pcrit; a higher Pcrit denotes that the organism requires a higher oxygen concentration for breathing (**A**). Pcrit generally increases monotonically with temperature (dashed line in **B**) because oxygen demand exceeds supply capacity. Endress and colleagues show, however, that some species display a U-shaped curve (solid line in **B**), suggesting that low temperatures also constrain oxygen uptake via reducing ventilatory capacity. This curve results in an optimal temperature at which aerobic capacity is maximum.

To investigate the interplay between metabolism, oxygen availability, and temperature, researchers typically measure parameter Pcrit, defined as the lowest oxygen partial pressure required to sustain resting metabolism [8]. When the availability of oxygen in the environment falls below the organism’s Pcrit, metabolic rates decline suddenly (Fig 1A). Empirical measurements suggest that Pcrit generally increases in relation to temperature (Fig 1B). As temperature rises, oxygen uptake and transport systems are unable to incorporate oxygen at the rate at which metabolizing tissues are burning it, suggesting that oxygen demand increases faster than supply and ultimately exceed it at high temperatures. In their study, Endress and colleagues show a striking pattern for Pcrit in different cold-blooded animals: They found that Pcrit displays a U-shaped curve—rather than a monotonic increase—in relation to temperature, suggesting that metabolic rates are oxygen limited, not only at warm but also at cold temperatures. But what causes this pattern?

To explain this U-shaped curve, Endress and colleagues simulated the chain of physical, chemical, and physiological processes involved in the transportation of oxygen molecules from the environment to the metabolizing body tissues. This chain includes external ventilation of water from environment to the gill surface, the molecular oxygen diffusion across that surface, and the internal flux of oxygen mediated by the circulatory system. Although every step in this chain responds to temperature due to physicochemical properties of their components, not all of them respond in the exact same way. Thus, the authors propose that the different thermal sensitivities of ventilation, circulation, diffusion, and metabolic demand led to complex temperature-dependent relationships of Pcrit: As temperature increases, the demand for oxygen increases faster than diffusion rates, whereas at cold temperatures, ventilatory or circulatory capacity slows down faster than oxygen demand. As a result, demand exceeds supply at both warm and cold extremes (increasing Pcrit), giving rise to an optimal region at intermediate temperatures where the oxygen supply chain is most effective and can thus maximize metabolic activity.

Climate—and particularly temperature—constrain species’ geographical ranges through its effect on physiological performance. Endress and colleagues’ mechanistic framework generates a biogeographic prediction: Species should live where temperature and oxygen availability allow maximizing metabolic activity. By comparing their predictions with empirical data on the geographical distribution of different marine species, the authors find support for this hypothesis. This idea provides new opportunities to examine the role of temperature and oxygen limitation in determining the geographical range of marine species, especially at their cold range limits and their responses to climate warming. These results also point to oxygen limitation as a mechanism explaining the decline in marine biodiversity towards the poles, where environmental conditions impose long-term aerobic constraints on physiology, and suggest that climate warming will alleviate this barrier facilitating latitudinal range expansions towards the poles.

Mechanistic models are breaking new ground in understanding and predicting the responses of organisms to climate change [9]. Endress and colleagues’ model makes a key contribution to this end, as it has the potential to capture the mechanisms limiting, not only the hot but also the cold edges of species distributions. Further research should address whether U-shaped Pcrit temperature-dependent curves are widespread in nature and, if they are, whether oxygen limitation is the most important cause determining these responses.
